# Construction of a molecular inflammatory predictive model with histone modification-related genes and identification of CAMK2D as a potential response signature to infliximab in ulcerative colitis

**DOI:** 10.3389/fimmu.2023.1282136

**Published:** 2024-01-11

**Authors:** Shuyu Ye, Yongqing Lyu, Libin Chen, Yiwei Wang, Yue He, Quansi Li, Li Tian, Fen Liu, Xiaoyan Wang, Feiyan Ai

**Affiliations:** ^1^ Department of Gastroenterology, The Third Xiangya Hospital of Central South University, Changsha, China; ^2^ Hunan Key Laboratory of Non-Resolving Inflammation and Cancer, The Third Xiangya Hospital, Central South University, Changsha, China; ^3^ Center for Medical Genetics and Hunan Key Laboratory of Medical Genetics, School of Life Sciences, Central South University, Changsha, Hunan, China; ^4^ Xiangya School of Medicine, Central South University, Changsha, Hunan, China

**Keywords:** histone modification, ulcerative colitis, molecular inflammatory predictive model, infliximab response, CAMK2D

## Abstract

**Background:**

Ulcerative colitis (UC) is a lifelong inflammatory disease affecting the rectum and colon with numerous treatment options that require an individualized treatment plan. Histone modifications regulate chromosome structure and gene expression, resulting in effects on inflammatory and immune responses. However, the relationship between histone modification-related genes and UC remains unclear.

**Methods:**

Transcriptomic data from GSE59071 and GSE66407 were obtained from the Gene Expression Omnibus (GEO), encompassing colonic biopsy expression profiles of UC patients in inflamed and non-inflamed status. Differentially expressed gene (DEG) analyses, functional enrichment analyses, weighted gene co-expression network analysis (WGCNA), and random forest were performed to identify histone modification-related core genes associated with UC inflammation. Features were screened through the least absolute shrinkage and selection operator (LASSO) and support vector machine‐recursive feature elimination (SVM‐RFE), establishing a molecular inflammatory predictive model using logistic regression. The model was validated in the GSE107499 dataset, and the performance of the features was assessed using receiver operating characteristic (ROC) and calibration curves. Immunohistochemistry (IHC) staining of colonic biopsy tissues from UC patients treated with infliximab was used to further confirm the clinical application value. Univariate logistic regression on GSE14580 highlighted features linked to infliximab response.

**Results:**

A total of 253 histone modification-related DEGs were identified between inflammatory and non-inflammatory patients with UC. Seven key genes (IL-1β, MSL3, HDAC7, IRF4, CAMK2D, AUTS2, and PADI2) were selected using WGCNA and random forest. Through univariate logistic regression, three core genes (CAMK2D, AUTS2, and IL-1β) were further incorporated to construct the molecular inflammatory predictive model. The area under the curve (AUC) of the model was 0.943 in the independent validation dataset. A significant association between CAMK2D protein expression and infliximab response was observed, which was validated in another independent verification set of GSE14580 from the GEO database.

**Conclusion:**

The molecular inflammatory predictive model based on CAMK2D, AUTS2, and IL-1β could reliably distinguish the mucosal inflammatory status of UC patients. We further revealed that CAMK2D was a predictive marker of infliximab response. These findings are expected to provide a new evidence base for personalized treatment and management strategies for UC patients.

## Introduction

Ulcerative colitis (UC) is a subtype of inflammatory bowel disease (IBD) characterized by recurrent and remitting chronic superficial mucosal ulceration and inflammation of the colon and rectum. The specific etiology of UC remains incompletely understood, while genetic susceptibility, immunological factors, and multiple environmental factors seem to contribute to the development of UC (1). The conventional therapies for ulcerative colitis include aminosalicylates, corticosteroids, and immunomodulators. A series of advanced treatments such as biologics and small-molecule drugs have emerged along with increased knowledge of the pathogenesis underlying UC. Nevertheless, the above available treatments remain unsatisfactory, possibly due to individual variations in patients’ intestinal inflammation molecular expression. For both patients and clinicians, selecting from various treatment options frequently presents a clinical challenge. Prior studies indicate that intestinal inflammation of patients with UC is associated with long-term outcomes and drug response (2, 3). Disease molecular measures may augment current endpoints and provide a more profound understanding of the disease mechanism (4). Therefore, the exploration of a molecular predictive model for inflammation might facilitate the customization of patient treatments.

Histone modifications regulate chromatin states and gene transcription, including histone acetylation, methylation, and phosphorylation. Emerging evidence suggests that patients with UC exhibit aberrant histone modifications accompanying inflammatory activity (5). Compared to healthy individuals, histone modification levels have been altered in the inflamed colonic mucosa of UC patients (6). Furthermore, increased histone acetylation at the promoters of pro-inflammatory cytokine genes, such as tumor necrosis factor-alpha (TNF-α) and interleukin-1β (IL-1β), has been associated with their upregulation in UC (7, 8). Similarly, altered histone methylation patterns have been linked to dysregulated expression of genes involved in immune cell activation and tissue damage (9, 10). Hence, exploring potential molecular biomarkers could provide insights into disease activity, and histone modifications have emerged as a promising avenue in this regard.

Biologic agents are crucial therapeutic options for patients with moderate-to-severe UC, and infliximab ranked first with the highest evidence for efficacy in inducing endoscopic improvement (11, 12). However, some patients show primary non-response or secondary loss of response to infliximab (13). Numerous studies have demonstrated that therapeutic drug and anti-drug antibody concentrations, clinical characteristics, mucosal immune features, gut microbial dysbiosis, and genetic factors are predictors of infliximab response (14–19). Notably, the anti-inflammatory effect of butyrate primarily arises from the activity of histone deacetylase inhibitors (20). Infliximab alleviates inflammation by binding to TNF-α. Similarly, histone modification could affect the expression of TNF-α and regulate the function of immune cells and anti-inflammatory pathways. It implied a possible synergistic effect of histone modification in the efficacy of infliximab treatment, making it a promising therapeutic target for UC (21). However, limited research has investigated the significance of histone modifications in forecasting inflammation and infliximab response among individuals with ulcerative colitis.

To address this gap in knowledge, the gene expression profiles of inflamed and non-inflamed colonic biopsies were retrieved from UC patients using the Gene Expression Omnibus (GEO) database, and histone modification-associated differentially expressed genes (DEGs) were identified using Wilcox analysis. Weighted gene co-expression network analysis (WGCNA) was employed to identify co-expressed gene modules. The key module eigengenes were functionally annotated through Gene Ontology (GO) and Kyoto Encyclopedia of Genes and Genomes (KEGG) enrichment analyses. Key genes were then selected using the random forest and support vector machine‐recursive feature elimination (SVM‐RFE) algorithms. Finally, a logistic regression model was constructed using the key genes for prediction purposes. The understanding of the gene expression regulation and immune response influences of histone modifications is expected to develop new therapeutic strategies for personalized approaches to UC management.

## Materials and methods

### Data selection and processing

Public data and full clinical annotation were downloaded from the GEO database. Combining GSE59071 and GSE66407 as the training cohort, the data standardization process was carried out before analysis, including data alignment, missing value processing, and probe annotation. When multiple probes corresponded to the same gene, the average value was selected as the expression level of the gene. Inflamed and non-inflamed colonic biopsies from UC patients were selected for the following analysis. Using the “sva” package to reduce the batch effect in different independent cohorts, GSE107499 and GSE14580 were selected as the test cohorts for the molecular inflammatory predictive model and infliximab response validation, respectively (**Table 1**).

**Table 1:** Brief descriptions of the inclusive data series.

	Platform	Data	Sample	Diagnosis	Active/response	Inactive/non-response	Cohort
Model							
GSE59071	GPL6244	Microarray	Biopsy	UC	74	23	Discovery
GSE66407	GPL19833	Microarray	Biopsy	UC	62	94	Discovery
GSE107499	GPL15207	Microarray	Biopsy	UC	75	44	Validation
Infliximab response							
GSE14580	GPL570	Microarray	Biopsy	UC	8	16	Validation

UC, ulcerative colitis.

### Screening for histone modification-related DEGs

In the training dataset, samples were categorized into UC_noninflammation (n = 117) and UC_inflammation (n = 136) groups. Histone modification-associated DEGs were identified using Wilcox analysis, setting an adjusted *p*-value threshold of <0.05. The significance criteria for DEGs were established accordingly. A volcano plot was generated using the R package “EnhancedVolcano”, while the heatmap of histone modification-related DEGs (22) was created using the R package “pheatmap”.

### WGCNA and functional enrichment analysis

WGCNA identified co-expressed gene modules, exploring gene network–phenotype relationships and key network genes. Using the “WGCNA” R package, histone modification-related genes were analyzed. Gene correlations were calculated, and weighted connections were established. A hierarchical clustering tree was constructed using correlation coefficients, indicating diverse gene modules through colors. The crucial module linked to inflammation was pinpointed, and module eigengenes were extracted for further analysis.

GO and KEGG pathway functional enrichment analyses of the module eigengenes were performed using the “clusterProfiler”, “org.Hs.eg.db”, “enrichplot”, and “ggplot2” packages in R software. Terms with *p* < 0.05 and q < 0.05 were considered significantly enriched.

### Select core histone modification-related DEGS

Random forest was used to screen key genes using the “randomforest” package. The error rate for 1–500 trees was calculated, and the lowest error rate and the best number of stable trees were determined. Further, the random forest method was used to screen the key genes, and the Gini coefficient method was used to calculate the dimensional significance value. Genes with a significant value greater than three were considered key genes. The intersection of the module eigengenes and the key genes were regarded as the core genes. Correlations between core genes were analyzed.

### Analysis of immune cell infiltration

We assessed the proportions of different infiltrating immune cell types between the UC_inflammation group and UC_noninflammation group using the CIBERSORT algorithm. We used LM22 and CIBERSORT matrices to predict the proportion of 22 infiltrating immune cell subtypes in a single sample of the dataset (23). We identified differentially enriched immune cells between UC_inflammation and UC_noninflammation samples and analyzed their correlation with core genes.

### Construction of a molecular inflammatory predictive model

Univariate logistic regression was used to screen core genes significantly associated with inflammation. To avoid model overfitting and filter out the most important events, the “glmnet” package in R was used to screen the most significant predictors using the least absolute shrinkage and selection operator (LASSO) regression (24). Meanwhile, an SVM‐RFE model was established using the “e1071” package in R, which was compared by the average misjudgment rates of their fivefold cross‐validations. Furthermore, a multivariate logistic regression model based on the most important predictors was constructed. For univariate and multivariate logistic regression analyses, *p*-value <0.05 was considered the cutoff for the significant correlation.

### Nomogram plot and calibration curve

A nomogram was established for a better clinical application of the model (25). A calibration curve was constructed using the “rms” package in R to compare the difference between the model-predicted probability and the actual probability.

### Verification using validation datasets

On the validation dataset (GSE107499), the multivariate logistic regression model was tested for effectiveness verification. The area under the curve (AUC) was calculated using the “ROCR” package in R. Univariate logistic regression was used on the validation dataset (GSE14580) to screen the core genes significantly associated with infliximab response.

### Clinical specimens and immunohistochemistry

Clinical data and endoscopic colon biopsy paraffin sections from February 2020 to December 2022 from 40 UC patients in the Third Xiangya Hospital were retrospectively collected. The inflamed (n = 20) and non-inflamed (n = 20) lesions were respectively obtained from the endoscopic activity and remission patients evaluated using Mayo endoscopic subscore from colonoscopy reports (26). The 20 patients presenting with endoscopic inflammation at baseline underwent subsequent treatment with infliximab. They were followed up for 8 weeks, and the infliximab response was assessed based on the Mayo endoscopic subscore. Ethical approval for the current study was granted by the institutional review board (IRB) of the Third Xiangya Hospital, Central South University (No. E.23188). More available details of these patients are depicted in **Supplementary Table S1**.

Paraffin sections were dewaxed, rehydrated, and incubated with 3% hydrogen peroxide for 15 minutes to block endogenous peroxidase. Then, sections were placed in 0.01 mol/L sodium citrate buffer for antigen repair, followed by washing with phosphate-buffered saline (pH = 7.4) two times for 5 minutes each and blocking with normal goat serum for 30 minutes. Immunostaining was performed by incubating with anti-CAMK2D antibody (1:100, DF12015, Affinity Biosciences, Jiangsu, China) or anti-IL-1β antibody (1:80, AF06643, AiFang biological, Hunan, China) at 4°C overnight. Sections were then washed in phosphate-buffered saline with Tween three times for 5 minutes each and incubated with secondary antibody for 30 minutes at room temperature. Bound antibodies were detected using diaminobenzidine, and slides were counterstained with hematoxylin.

A semi-quantitative scoring system was used to assess the expression of CAMK2D and IL-1β (protein expression = expression intensity + expression area). Expression intensity was scored using a 4-point scale from 0 to 3. Expression area according to the percentage of positive-stained cells was scored using a 5-point scale from 0 to 4. Finally, the summation scores divided each slide into low expression (0–2) and high expression (3–7). These results were confirmed by two independent pathologists who were blinded to patient clinical data.

### Statistical analysis

All the statistical analyses and visualization of the GEO set samples were performed using R 4.1.1. A two-sided *p*-value of <0.05 was considered statistically significant unless otherwise noted.

## Results

### Screening for histone modification-related DEGs

**Figure 1:**
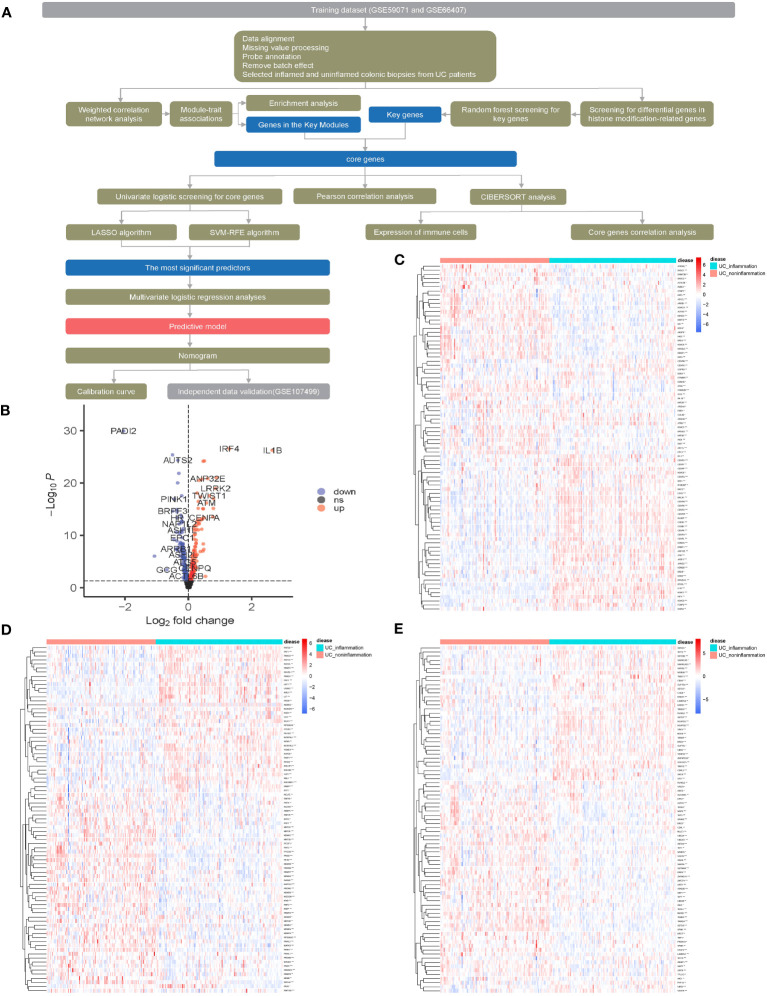
Screening for histone modification-related differentially expressed genes (DEGs) between inflamed and non-inflamed tissues of ulcerative colitis patients. **(A)** Flowchart of the dataset analysis. **(B)** Volcano plot and **(C**–**E)** heatmap of histone modification-related DEGs between inflamed and non-inflamed tissues.

The flowchart of the dataset analysis is described in **Figure 1A**. We merged the GSE59071 and GSE66407 datasets and removed batch effects. To further explore DEGs between inflamed and non-inflamed tissues, we conducted a differential analysis of 253 patients diagnosed with ulcerative colitis and 431 histone modification-related genes. We identified a total of 253 histone modification-related DEGs, including 129 downregulated genes and 124 upregulated genes (**Figures 1B–E**; **Supplementary Table S2**).

### Selecting key DEGs related to inflammation of UC

**Figure 2:**
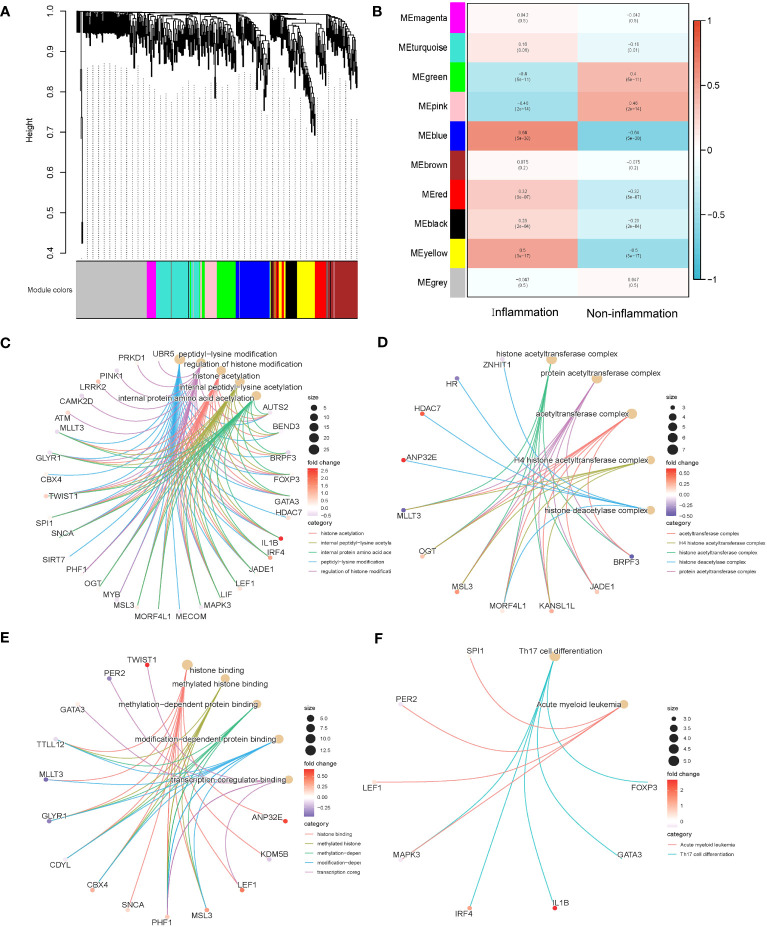
Co-expression and functional enrichment analysis of histone modification-related differentially expressed genes. **(A)** Gene dendrogram and module colors. **(B)** Correlation results between the 10 modules and two inflammatory states. **(C)** GO functional enrichment analysis of the intersecting genes with BP terms in the blue module. **(D)** GO functional enrichment analysis of the intersecting genes with CC terms in the blue module. **(E)** GO functional enrichment analysis of the intersecting genes with MF terms in the blue module. **(F)** KEGG annotation map of genes in the blue module. GO, Gene Ontology; BP, biological process; CC, cellular component; MF, molecular function; KEGG, Kyoto Encyclopedia of Genes and Genomes.

**Figure 3:**
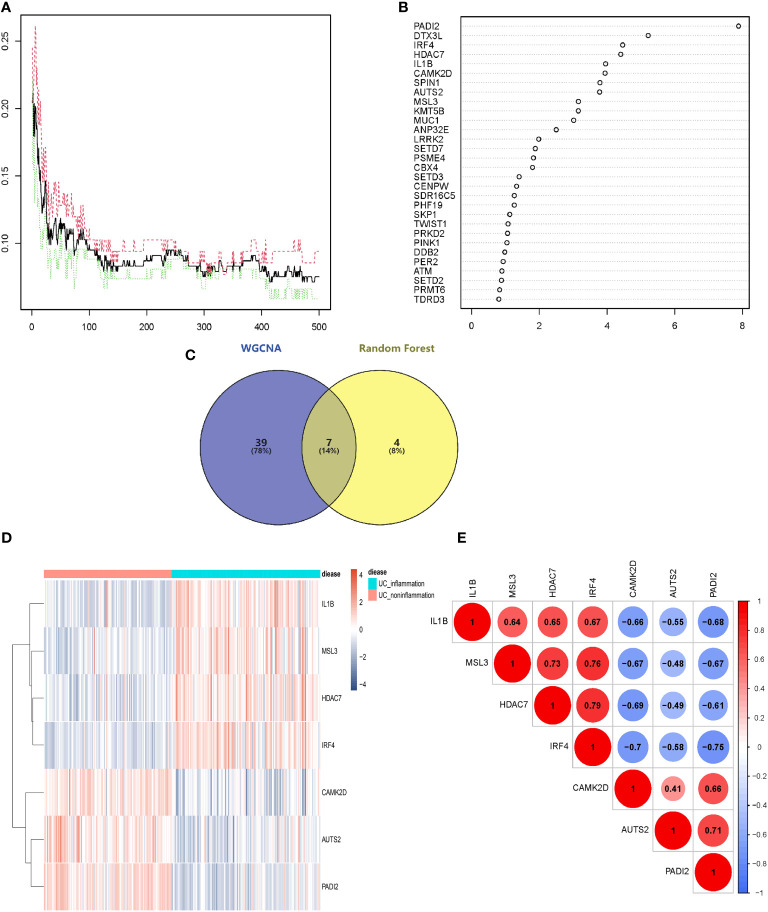
Selecting core histone modification-related differentially expressed genes. **(A)** The lowest error rate and the best number of stable trees were determined. **(B)** The top 11 significant genes were screened (seed = 213, MeanDecreaseGini > 3). **(C)** Venn diagram indicating intersections of leading genes in WGCNA and top significant genes in random forest. **(D)** Heatmap to explore the correlations between core genes and inflammatory status of UC. **(E)** Correlation analysis of seven core histone modification-related genes. UC, ulcerative colitis; WGCNA, weighted gene co-expression network analysis.

The above 253 histone modification-related DEGs were classified into 10 modules by hierarchical clustering tree (**Figures 2A, B**). The blue module was identified as having the most significant positive correlation with inflammation (0.64) related to ulcerative colitis. GO and KEGG functional enrichment analyses on the 46 genes contained in the blue module were performed. A total of 31 genes were identified to be significantly enriched in pathways related to biological processes, 11 genes in pathways related to cellular components, and 14 genes in pathways related to molecular function (*p* < 0.05 and q < 0.05). The top five biological processes, cellular components, and molecular function pathways are shown in **Figures 2C–E**, respectively. The KEGG annotation results indicated eight genes significantly enriched in Th17 cell differentiation and acute myeloid leukemia pathways (**Figure 2F**). The lowest error rate and the best number of stable trees were determined using random forest (**Figure 3A**). The most contributable 11 genes were intersected in the random forest (MeanDecreaseGini > 3) and the significant 46 module eigengenes in WGCNA (**Figures 3B, C**). Seven genes (IL-1β, MscS-Like 3 [MSL3], histone deacetylase 7 [HDAC7], interferon regulatory factor 4 [IRF4], calcium/calmodulin-dependent kinase II-delta [CAMK2D], autism susceptibility candidate 2 [AUTS2], and peptidyl arginine deiminase 2 [PADI2]) were assessed as the core genes. Higher expression of IL-1β, MSL3, HDAC7, and IRF4 and lower expression of CAMK2D, AUTS2, and PADI2 were correlated with inflammation in UC patients (**Figure 3D**). Further correlation analysis revealed a universal correlation between core histone modification-related genes in UC inflammation (**Figure 3E**).

### Analysis of immune cell infiltration

**Figure 4:**
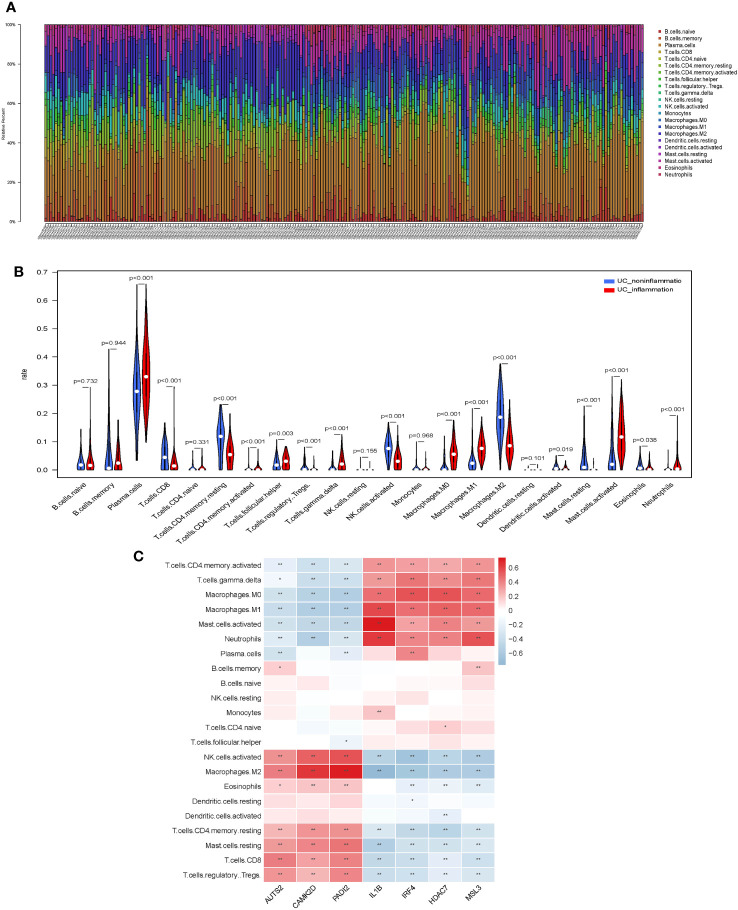
Immunological features of core histone modification-related genes in UC inflammation. **(A)** Bar plot of percentage infiltration of 22 immune cells in each sample. **(B)** Differential infiltration of the 22 immune cells in UC patients with endoscopic inflamed and non-inflamed conditions. **(C)** Correlation heatmap of 22 immune cell types with seven core genes. UC, ulcerative colitis. Asterisks represent levels of significance (*p < 0.05, **p < 0.01).

We evaluated the distribution and calculated the immune cell proportion of 22 immune cell types using CIBERSORT (**Figures 4A, B**). Significant differences in the proportion of immune cell features were exhibited between inflamed and non-inflamed UC patients, including plasma cells, CD8^+^ T cells, CD4^+^ memory resting T cells, CD4^+^ memory activated T cells, follicular helper T cells, regulatory T cells, γδ T cells, activated NK cells, M0 macrophages, M1 macrophages, M2 macrophages, activated dendritic cells, resting mast cells, activated mast cells, eosinophils, and neutrophils. The above immune cells may play an important role in the inflammatory process of UC.

Subsequently, we assessed the correlation between core histone modification-related genes and immune cells in UC inflammation (**Figure 4C**). Activated NK cells, M2 macrophages, eosinophils, CD4^+^ memory resting T cells, resting mast cells, CD8^+^ T cells, and regulatory T cells were more abundant in the non-inflammatory UC patients and positively correlated with AUTS2, CAMK2D, and PADI2. On the contrary, CD4^+^ memory-activated T cells, γδ T cells, M0 macrophages, M1 macrophages, activated mast cells, and neutrophils were more abundant in the inflammatory UC patients and positively correlated with IL-1β, IRF4, HDAC7, and MSL3.

### Constructing and validating the molecular inflammatory predictive model

**Figure 5:**
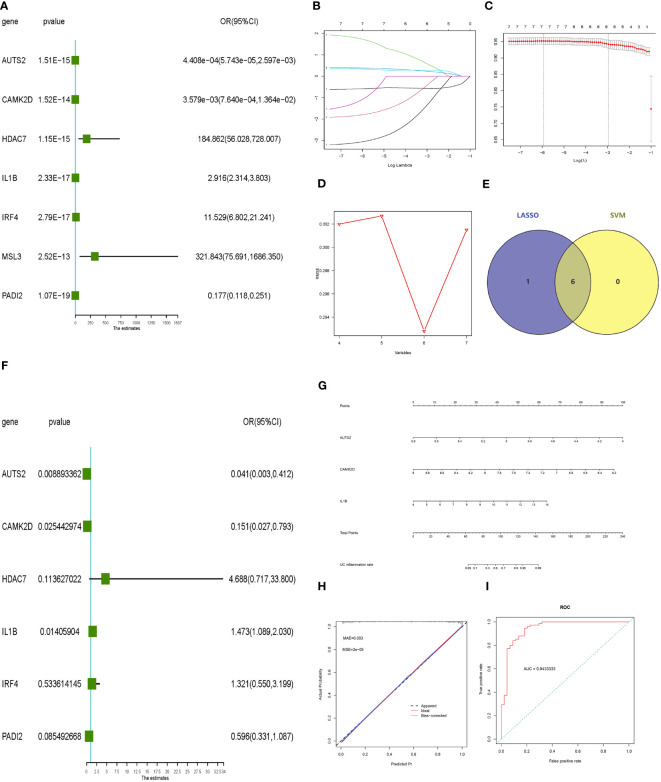
Constructing and validating the inflammatory predictive model. **(A)** Forest plot lines of significant core genes related to UC inflammation using univariate logistic regression. **(B, C)** LASSO regression and **(D)** SVM‐RFE model of the combined training dataset. **(E)** Venn diagram of core genes related to UC inflammation between LASSO analysis and SVM‐RFE model. **(F)** Forest plot lines of significant core genes related to UC inflammation using multivariate logistic regression. **(G, H)** Nomogram plot and calibration curve of the inflammatory predictive model. **(I)** Evaluation of the inflammatory predictive model in the validation dataset. UC, ulcerative colitis; LASSO, least absolute shrinkage and selection operator; SVM‐RFE, support vector machine‐recursive feature elimination.

Univariate logistic regression, LASSO regression, and the SVM‐RFE model were performed to screen potential inflammatory predictive markers from core histone modification-related genes in UC inflammation (**Figures 5A–D**). As shown in **Figures 5E, F**, three core genes (AUTS2, CAMK2D, and IL-1β) were further applied in multivariate logistic regression. A nomogram model was established (**Figure 5G**), and the C-index value of the model was 0.948. Calibration curves showed the accuracy of this model in predicting the inflammation rate of UC patients (**Figure 5H**). The inflammatory predictive model was validated on the GSE107499 dataset, and the AUC value was 0.943, which showed that the model had high accuracy in predicting the inflammatory status of UC patients (**Figure 5I**).

### Predictive gene validation in UC patients and infliximab response

**Figure 6:**
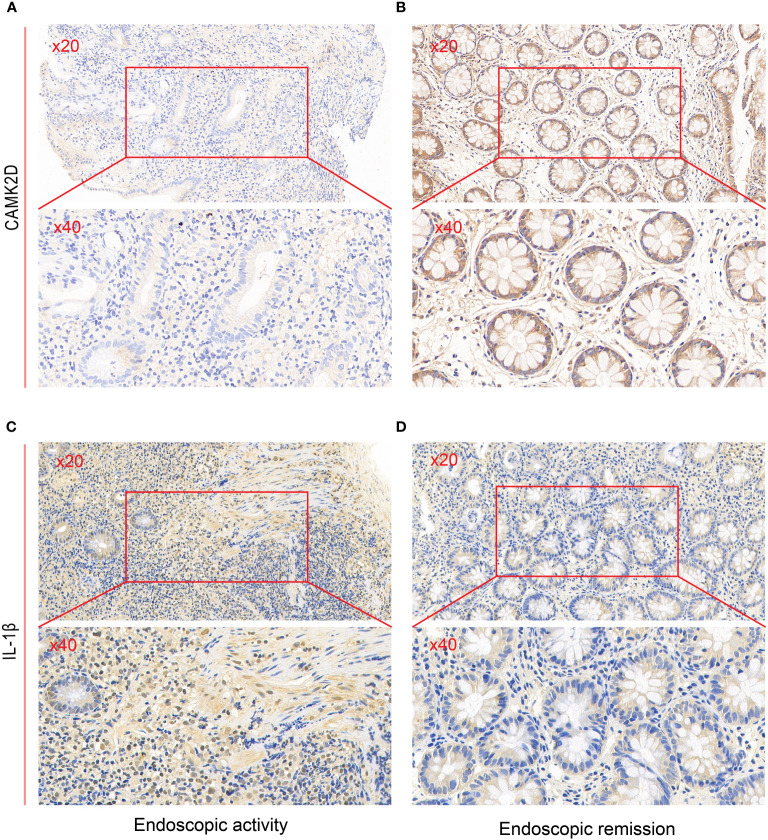
Validating predictive genes (IL-1β and CAMK2D) by IHC in UC patients. IHC staining of CAMK2D in **(A)** endoscopic activity and **(B)** endoscopic remission patients. IHC staining of IL-1β in **(C)** endoscopic activity and **(D)** endoscopic remission patients. UC, ulcerative colitis; IHC, immunohistochemistry.

We performed immunohistochemistry to detect the relationship between endoscopic inflammatory status and CAMK2D protein expression. We leveraged clinical data on erythrocyte sedimentation rate (ESR) from the electronic medical record system of the Third Xiangya Hospital, which is a common clinical inflammation indicator for disease diagnosis and management of UC patients. The level of ESR was lower in patients with higher CAMK2D expression (**Supplementary Figure S1**). A negative correlation between the histology severity of colon biopsies and CAMK2D protein expression level was found (*p* = 0.0024). To our knowledge, IL-1β is a traditional inflammatory cytokine and has been reported as highly expressed in UC patients. As shown in **Figures 6A–D**, CAMK2D protein expression was negatively correlated with endoscopic inflammation, whereas IL-1β protein expression was positively correlated with endoscopic inflammation. These results indicated that CAMK2D worked as an anti-inflammation molecule in UC.

We further followed up on 20 UC patients with endoscopic inflammation at baseline and initiated infliximab treatment for 8 weeks. Patients with high CAMK2D protein expression were associated with infliximab response (**Supplementary Figure S2**). We validated the result in GSE14580 (**Supplementary Figure S3**), which indicated a predictive role of CAMK2D in infliximab response.

## Discussion

Over the past two decades, the goal in treating IBD has shifted from symptom relief to achieving consistent clinical and endoscopic remission to halt the natural disease progression and reduce the risk of complications and surgery; however, the rate is not encouraging (27). A meta-analysis reported that UC patients with endoscopic remission after initiation of therapy were associated with improved long-term outcomes such as corticosteroid-free clinical and endoscopic remission (28). Long-term and persistent colonic inflammation in UC increases the risk of relapse, steroid dependency, colectomy, and even colorectal cancer (29, 30). Therefore, it is important to focus on endoscopic inflammation status at baseline.

A range of inflammation biomarkers were upregulated in UC, which exacerbates chronic colon inflammation and mucosal damage, such as TNF-α, IL-1β, IL-6, and IFN-γ. Histone acetylation promotes these pro-inflammatory gene expressions and inflammasome activation (7, 8). Evidence showed that aberrant histone acetylation exists in UC patients (6) and colitis mice colon (31), while using histone deacetylase (HDAC) inhibitors could attenuate colitis by suppressing pro-inflammatory cytokines (32, 33), acetylating transcription of STAT1 and NF-κB (34, 35), increasing the formation of IL-35 *via* EBI3 (36), interacting with non-coding RNAs to fine-tune inflammatory responses (37), etc. Additionally, aberrant histone methylation patterns contribute to dysregulated inflammation (38, 39). Therefore, targets for histone modification may be potential candidate treatments for UC patients with endoscopic inflammation (21, 40). Machine learning algorithms are increasingly being used to create decision models that aid in disease diagnosis and treatment. In the current study, we identified histone modification-related inflammatory genes of UC within endoscopic activity versus endoscopic remission groups through integrated bioinformatics analysis, suggesting a key role of IL-1β, AUTS2, and CAMK2D in UC endoscopic inflammatory state. IL-1β is a classical pro-inflammatory cytokine regulated by histone modification that increases intestinal epithelial permeability, recruits neutrophils to inflamed colon tissue, and compromises the epithelial barrier (9, 10, 41, 42). Canakinumab treatment demonstrated significantly higher response rates in patients with very early onset IBD (43). Preclinical studies supported the effectiveness of anti-IL-1β monoclonal antibodies 7F IgG and FL-BsAb1/17 in mitigating colitis (44, 45), while anakinra showed efficacy in the UC model of primary anti-TNF non-responders (46). Targeting IL-1β is a potential therapeutic option for ameliorating UC. AUTS2, mainly expressed in the brain, is linked to various neurological diseases (47). A genome-wide association study reported a downregulated expression of AUTS2 in endoscopic pinch biopsies of Crohn’s disease (CD) patients compared to healthy individuals (48). CAMK2D has been implicated in the modulation of inflammatory processes. NF-κB regulates CAMK2D transcription, and CAMK2D protein modulates NF-κB signaling and leukocyte trans-endothelial migration to inflammatory sites (49–51). Therefore, histone modification may play an important role in UC, which needs to be explored further.

Emerging evidence suggests the innate and adaptive immune response plays a crucial role in inducing intestinal inflammation (52). We also identified significant differences in the proportions of some important immune cells such as plasma cells, T cells, NK cells, macrophages, neutrophils, and mast cells between inflamed and non-inflamed mucosa in UC patients using CIBERSORT. Mounting evidence reported the association between these immune cells and histone modification-related genes. Toll-like receptor (TLR) signaling, a key factor in the innate immune response, is characterized by histone modifications, which induce the expression of inflammation-related genes (53). *N*-Butyrate enhances histone acetylation in macrophages, downregulates pro-inflammatory mediators, and regulates B-lymphocyte differentiation (54). It helps maintain tolerance to commensal organisms by dampening colonic macrophage response (55). Moreover, monocytes differentiate into dendritic cells through CD14 and CD209 gene regulation, influenced by histone modifications, impacting IBD pathogenesis (56, 57). HDAC6 inhibitor may attenuate colonic inflammation by reducing the activation of neutrophils (58). Additionally, IBD patients exhibit hyperactive effector CD4^+^ T cells and an imbalance of regulatory T (Treg) and Th17 cells associated with histone deacetylase and methylation (59–62). EZH2, a histone methyltransferase, decreased in IBD patients (63), and the deficiency in Treg cells induces pro-inflammatory cytokines and spontaneous IBD (64). The function of Treg cells in IBD patients has been reported to correlate with treatment response and can be regulated by HDAC inhibitors (18, 65). Consistent with the content reported in the above literature, our data showed that the abundance of macrophages, monocytes, neutrophils, and T cells was associated with histone modification-related genes, such as IL-1β, AUTS2, and CAMK2D. These elements contribute to the importance of immune dysfunction and histone modifications in UC pathogenesis.

We conducted validation on two independent datasets to ensure the robustness of our results. First, we utilized the validation dataset GSE107499 to assess the accuracy of the predictive model that we developed. The model demonstrated excellent performance on this validation dataset as well (AUC > 0.9), indicating that AUTS2, CAMK2D, and IL-1β are indeed potential diagnostic markers capable of distinguishing between endoscopic inflammatory and non-inflammatory UC patients. Subsequently, we followed up 20 UC patients treated with infliximab and revealed an association between CAMK2D high expression and infliximab response, which was further validated in an external dataset. CAMK2D has been reported to be involved in several classical inflammatory pathways associated with UC, including NF-κB, STAT3, IL-6, and TNF-α (50, 66, 67). Dahlen et al. demonstrated lower mucosal IFN-γ, IL-1β, IL-6, and TNF-α mRNA expression in UC patients with anti-TNF response (68). A model based on these serum cytokines can classify UC patients as primary infliximab responders and non-responders (69). Notably, CAMK2D is an IFN-γ-induced gene calculated as a substitute indicator for IFN-γ level, emphasizing its potential in assessing immune responses (70). The findings suggested that CAMK2D may partially explain the poorer immune response in patients with endoscopic inflammatory states and may serve as a predictive marker of infliximab treatment response.

However, there were several limitations to our study. First, our study primarily relied on bioinformatics analysis and limited clinical validation. While we validated the results in different databases, potential bias from different microarray platforms, RNA extraction methods, statistical methods, and limited clinical samples could not be avoided. Thus, more experimental and clinical studies are needed to further prove the current findings. Moreover, we lacked detailed *in vitro* and *in vivo* experiments to elucidate the specific mechanisms underlying histone modification genes, particularly CAMK2D, in relation to infliximab response and endoscopic inflammatory status. Finally, due to the COVID-19 pandemic, declined enrollment (71), and limited colonic biopsy, we lacked participants treated with other biologic agents and small-molecule drugs. Therefore, further research is necessary to explore the predictive function of CAMK2D for other treatments.

To conclude, our study identified pivotal molecular mechanisms and potential therapeutic implications of targeting histone modifications for UC. We identified and validated three histone modification-DEGs related to endoscopic inflammatory status. Moreover, CAMK2D has been preliminarily identified as a key predictive biomarker of response to infliximab, potentially exerting its influence on progression through histone modifications. These findings are expected to provide a new evidence base for personalized treatment and management strategies for UC patients.

## Data availability statement

The original contributions presented in the study are included in the article/**Supplementary Material**. Further inquiries can be directed to the corresponding author.

## Ethics statement

The studies involving humans were approved by The Ethics Committee of China’s Third Xiangya Hospital. The studies were conducted in accordance with the local legislation and institutional requirements. The human samples used in this study were acquired from a by-product of routine care or industry. Written informed consent for participation was not required from the participants or the participants’ legal guardians/next of kin in accordance with the national legislation and institutional requirements.

## Author contributions

SY: Conceptualization, Methodology, Formal analysis, Writing – original draft. YL: Conceptualization, Formal analysis, Methodology, Writing – original draft. LC: Methodology, Writing – review & editing. YW: Methodology, Writing – original draft. YH: Formal analysis, Writing – review & editing. QL: Writing – review & editing, Writing – original draft. LT: Writing – review & editing, Methodology. FL: Methodology, Writing – review & editing. XW: Writing – review & editing, Conceptualization, Formal analysis. FA: Conceptualization, Writing – review & editing, Methodology, Project administration.
