# Supplementary material for: Construction of a molecular inflammatory predictive model with histone modification-related genes and identification of CAMK2D as a potential response signature to infliximab in ulcerative colitis
Source: Front Immunol. 2024 Jan 11;14:1282136. doi: 10.3389/fimmu.2023.1282136 (PMC10808628; doi:10.3389/fimmu.2023.1282136)
Supplement: Supplementary file 1 [file DataSheet_1.docx]

## Supplementary Table S1. Clinical information of 40 colon specimens^1^.

| Number | Age | Gender | Biopsy position | Endoscopic status |
| --- | --- | --- | --- | --- |
| 1 | 21 | Male | Colon | Active |
| 2 | 31 | Female | Colon | Active |
| 3 | 31 | Female | Colon | Active |
| 4 | 32 | Male | Colon | Active |
| 5 | 40 | Female | Colon | Active |
| 6 | 41 | Male | Colon | Active |
| 7 | 42 | Female | Colon | Active |
| 8 | 43 | Female | Colon | Active |
| 9 | 45 | Male | Colon | Active |
| 10 | 45 | Male | Colon | Active |
| 11 | 50 | Female | Colon | Active |
| 12 | 52 | Male | Colon | Active |
| 13 | 52 | Female | Colon | Active |
| 14 | 55 | Female | Colon | Active |
| 15 | 56 | Male | Colon | Active |
| 16 | 57 | Female | Colon | Active |
| 17 | 58 | Female | Colon | Active |
| 18 | 59 | Male | Colon | Active |
| 19 | 60 | Male | Colon | Active |
| 20 | 64 | Female | Colon | Active |
| 21 | 21 | Male | Colon | Remission |
| 22 | 32 | Female | Colon | Remission |
| 23 | 35 | Male | Colon | Remission |
| 24 | 40 | Female | Colon | Remission |
| 25 | 42 | Female | Colon | Remission |
| 26 | 42 | Female | Colon | Remission |
| 27 | 42 | Male | Colon | Remission |
| 28 | 43 | Female | Colon | Remission |
| 29 | 45 | Male | Colon | Remission |
| 30 | 48 | Male | Colon | Remission |
| 31 | 50 | Female | Colon | Remission |
| 32 | 51 | Male | Colon | Remission |
| 33 | 53 | Female | Colon | Remission |
| 34 | 55 | Female | Colon | Remission |
| 35 | 56 | Male | Colon | Remission |
| 36 | 57 | Male | Colon | Remission |
| 37 | 58 | Male | Colon | Remission |
| 38 | 58 | Female | Colon | Remission |
| 39 | 63 | Female | Colon | Remission |
| 40 | 68 | Female | Colon | Remission |

## ^1^ Endoscopic status was evaluated by Mayo endoscopic subscore.

**Supplementary Table S2. The** **histone modification-related** **differentially expressed genes (DEGs^1^) between inflamed and non-inflamed tissue of ulcerative colitis patients.**

| **Symbol** | ***P* value** | **Log_2_ Fold Change** | **Adjusted *P* value** |
| --- | --- | --- | --- |
| ACTL6B | 0.006263 | -0.07019 | 0.011073 |
| AKAP8 | 0.006069 | -0.04867 | 0.010829 |
| ANP32E | 1.41E-21 | 0.609862 | 5.47E-20 |
| ARID4A | 0.013251 | -0.12078 | 0.021477 |
| ARID4B | 0.002618 | 0.105268 | 0.005143 |
| ARRB1 | 3.85E-08 | -0.39196 | 1.78E-07 |
| ASH1L | 1.11E-11 | -0.2047 | 8.14E-11 |
| ASH2L | 5.07E-07 | -0.16561 | 1.84E-06 |
| ATG5 | 1.22E-05 | -0.11673 | 3.57E-05 |
| ATM | 6.29E-17 | 0.573378 | 1.11E-15 |
| ATRX | 4.68E-05 | 0.178909 | 0.000125 |
| ATXN3L | 0.002865 | -0.09007 | 0.005545 |
| AUTS2 | 4.43E-25 | -0.337 | 3.44E-23 |
| BEND3 | 9.94E-07 | -0.19138 | 3.42E-06 |
| BRCA1 | 6.78E-12 | 0.247968 | 5.17E-11 |
| BRD1 | 3.18E-05 | -0.10939 | 8.83E-05 |
| BRD8 | 0.028833 | 0.070728 | 0.044865 |
| BRPF3 | 2.34E-15 | -0.50024 | 3.25E-14 |
| CAMK2D | 4.16E-26 | -0.50269 | 4.05E-24 |
| CCNB1 | 7.12E-16 | 0.790104 | 1.15E-14 |
| CENPA | 4.25E-14 | 0.498439 | 4.86E-13 |
| CENPH | 0.00429 | 0.162348 | 0.007908 |
| CENPI | 6.52E-05 | 0.144838 | 0.000169 |
| CENPK | 4.36E-09 | 0.468056 | 2.38E-08 |
| CENPL | 2.27E-05 | 0.164687 | 6.45E-05 |
| CENPM | 8.06E-06 | 0.263348 | 2.41E-05 |
| CENPN | 6.73E-16 | 0.307949 | 1.14E-14 |
| CENPO | 1.11E-07 | 0.163743 | 4.54E-07 |
| CENPP | 4.83E-12 | 0.16423 | 3.88E-11 |
| CENPQ | 0.000177 | 0.203367 | 0.000421 |
| CENPU | 1.06E-07 | 0.454705 | 4.37E-07 |
| CENPW | 9.91E-18 | 0.79302 | 2.03E-16 |
| CENPX | 4.05E-07 | 0.202424 | 1.51E-06 |
| CHEK1 | 8.53E-14 | 0.439818 | 9.22E-13 |
| COPRS | 0.002801 | 0.133848 | 0.005476 |
| CTBP1 | 0.009436 | -0.04622 | 0.015753 |
| CTNNB1 | 0.000758 | 0.147097 | 0.001628 |
| CUL4B | 0.013379 | 0.079177 | 0.021595 |
| DDB1 | 0.001897 | 0.091192 | 0.003784 |
| DDB2 | 4.50E-13 | 0.323806 | 4.07E-12 |
| DMAP1 | 4.89E-12 | -0.15422 | 3.88E-11 |
| DNMT1 | 8.80E-16 | 0.466913 | 1.32E-14 |
| DNMT3B | 0.00239 | -0.04443 | 0.004719 |
| DTX3L | 7.06E-25 | 0.464513 | 3.92E-23 |
| DYDC1 | 5.55E-06 | -0.10556 | 1.69E-05 |
| DYDC2 | 0.000499 | -0.07669 | 0.001115 |
| EPC1 | 2.84E-10 | -0.19786 | 1.81E-09 |
| EZH2 | 6.08E-11 | 0.214269 | 4.01E-10 |
| FMR1 | 0.029087 | -0.06132 | 0.045078 |
| FOXP3 | 1.29E-11 | 0.199854 | 9.33E-11 |
| GATA3 | 0.003205 | 0.07908 | 0.006082 |
| GCG | 0.000377 | -0.681 | 0.000863 |
| H1.3 | 0.005123 | 0.12378 | 0.009313 |
| H2BC1 | 0.007243 | -0.0759 | 0.012522 |
| H4.16 | 0.001447 | -0.10132 | 0.002962 |
| H4C3 | 0.001821 | 0.132506 | 0.003652 |
| HAT1 | 1.12E-06 | 0.220886 | 3.83E-06 |
| HDAC11 | 3.26E-09 | -0.25115 | 1.86E-08 |
| HDAC5 | 2.48E-06 | -0.13602 | 7.83E-06 |
| HDAC6 | 0.000375 | -0.09507 | 0.000862 |
| HDAC7 | 6.11E-25 | 0.505393 | 3.92E-23 |
| HDAC8 | 0.009155 | 0.055359 | 0.015351 |
| HDAC9 | 4.48E-06 | 0.116413 | 1.37E-05 |
| HJURP | 6.23E-14 | 0.431018 | 6.92E-13 |
| HR | 4.14E-14 | -0.35197 | 4.86E-13 |
| HSF4 | 0.008067 | -0.05412 | 0.013763 |
| IL1B | 5.73E-27 | 2.655437 | 7.44E-25 |
| ING4 | 0.000173 | -0.13117 | 0.000418 |
| ING5 | 1.84E-06 | -0.10697 | 6.06E-06 |
| IRF4 | 2.81E-27 | 1.290363 | 5.47E-25 |
| ITGB3BP | 0.001544 | 0.12814 | 0.003145 |
| JADE1 | 4.99E-10 | 0.181376 | 3.13E-09 |
| JARID2 | 6.28E-05 | 0.151656 | 0.000164 |
| KANSL1 | 1.37E-06 | -0.13282 | 4.60E-06 |
| KANSL1L | 1.36E-12 | 0.257789 | 1.14E-11 |
| KANSL3 | 3.37E-07 | -0.12442 | 1.29E-06 |
| KAT2B | 0.000169 | 0.127173 | 0.000412 |
| KAT5 | 2.88E-06 | -0.14866 | 8.97E-06 |
| KAT6B | 1.73E-06 | -0.17347 | 5.76E-06 |
| KAT7 | 0.000311 | -0.14269 | 0.000725 |
| KAT8 | 3.78E-08 | -0.19996 | 1.77E-07 |
| KDM1B | 0.011677 | -0.10155 | 0.019085 |
| KDM2A | 2.93E-09 | 0.24449 | 1.70E-08 |
| KDM2B | 1.52E-08 | 0.223238 | 7.57E-08 |
| KDM3B | 1.89E-08 | -0.15772 | 9.31E-08 |
| KDM4A | 7.57E-07 | -0.26444 | 2.68E-06 |
| KDM4B | 0.016267 | 0.04536 | 0.025828 |
| KDM4C | 3.29E-08 | -0.18061 | 1.56E-07 |
| KDM5B | 5.40E-07 | -0.16726 | 1.94E-06 |
| KDM5C | 0.010271 | -0.06625 | 0.017002 |
| KDM5D | 0.006702 | 0.539301 | 0.011743 |
| KDM6A | 1.18E-09 | -0.36369 | 7.04E-09 |
| KDM7A | 1.94E-06 | -0.14886 | 6.33E-06 |
| KDM8 | 0.000842 | -0.11582 | 0.00179 |
| KMT2C | 2.26E-06 | -0.14268 | 7.19E-06 |
| KMT2D | 0.005206 | -0.06847 | 0.009375 |
| KMT2E | 1.85E-11 | -0.24289 | 1.31E-10 |
| KMT5B | 1.43E-22 | -0.30107 | 6.94E-21 |
| KNL1 | 8.83E-12 | 0.315438 | 6.61E-11 |
| LEF1 | 7.75E-16 | 0.458502 | 1.21E-14 |
| LIF | 8.24E-10 | 0.250402 | 5.01E-09 |
| LRRK2 | 1.12E-19 | 0.859579 | 3.12E-18 |
| MAPK3 | 2.09E-08 | -0.31294 | 1.02E-07 |
| MBIP | 6.02E-08 | -0.21984 | 2.66E-07 |
| MCM3AP | 0.006633 | 0.125274 | 0.011675 |
| MECOM | 2.12E-09 | -0.24958 | 1.25E-08 |
| MIS18A | 8.70E-05 | 0.178868 | 0.000223 |
| MIS18BP1 | 2.98E-06 | 0.195818 | 9.20E-06 |
| MORF4L1 | 0.000112 | 0.082028 | 0.000282 |
| MORF4L2 | 9.85E-14 | 0.301974 | 1.04E-12 |
| MSL3 | 3.16E-21 | 0.341735 | 1.02E-19 |
| MSL3P1 | 0.000535 | 0.185822 | 0.001183 |
| MTF2 | 0.01537 | -0.09655 | 0.024604 |
| MUC1 | 9.11E-22 | 0.879981 | 3.94E-20 |
| MYB | 4.37E-08 | -0.35997 | 2.00E-07 |
| MYOD1 | 5.55E-05 | -0.09231 | 0.000146 |
| NAA40 | 3.69E-05 | -0.17247 | 0.0001 |
| NAA50 | 0.025483 | 0.100742 | 0.039971 |
| NAP1L2 | 6.66E-13 | -0.28903 | 5.76E-12 |
| NCOA1 | 0.003996 | -0.10322 | 0.007438 |
| NELFE | 0.000616 | -0.09272 | 0.001353 |
| NOC2L | 0.000762 | 0.141645 | 0.00163 |
| NPM1 | 0.001619 | 0.140207 | 0.003264 |
| NR1H4 | 9.27E-07 | -1.0718 | 3.22E-06 |
| NSD1 | 0.001268 | 0.094592 | 0.002681 |
| OGT | 2.65E-05 | 0.202924 | 7.46E-05 |
| OIP5 | 4.72E-08 | 0.484417 | 2.14E-07 |
| OTUB1 | 0.004553 | 0.100747 | 0.008315 |
| PADI1 | 7.75E-05 | -0.07847 | 0.0002 |
| PADI2 | 1.18E-30 | -2.04973 | 4.60E-28 |
| PADI4 | 0.000748 | 0.186434 | 0.001617 |
| PAF1 | 5.79E-08 | 0.200992 | 2.59E-07 |
| PAX5 | 0.01689 | 0.011773 | 0.026709 |
| PCGF1 | 0.011908 | -0.10051 | 0.019381 |
| PER2 | 1.18E-15 | -0.3672 | 1.70E-14 |
| PHF1 | 1.35E-08 | 0.201605 | 6.89E-08 |
| PHF19 | 3.35E-17 | 0.312877 | 6.20E-16 |
| PHF2 | 2.38E-08 | -0.1638 | 1.14E-07 |
| PHF20 | 6.19E-08 | 0.168068 | 2.68E-07 |
| PHF8 | 0.001447 | -0.06932 | 0.002962 |
| PIH1D1 | 1.48E-05 | 0.108845 | 4.28E-05 |
| PINK1 | 1.19E-17 | -0.45336 | 2.31E-16 |
| PIWIL1 | 0.001339 | -0.12346 | 0.002785 |
| PIWIL2 | 6.50E-08 | -0.35204 | 2.78E-07 |
| PRDM12 | 0.000177 | -0.104 | 0.000421 |
| PRDM4 | 2.76E-11 | -0.20323 | 1.88E-10 |
| PRDM5 | 0.00032 | -0.0949 | 0.000741 |
| PRDM9 | 4.97E-05 | -0.09056 | 0.000132 |
| PRKD1 | 9.08E-05 | 0.225283 | 0.000231 |
| PRKD2 | 6.33E-13 | 0.34032 | 5.60E-12 |
| PRMT1 | 9.08E-11 | 0.177564 | 5.89E-10 |
| PRMT6 | 6.08E-08 | -0.22323 | 2.66E-07 |
| PRMT7 | 0.000129 | -0.1256 | 0.00032 |
| PRMT8 | 0.002011 | -0.06097 | 0.00399 |
| PSME4 | 4.46E-18 | 0.383015 | 9.64E-17 |
| PWP1 | 1.46E-05 | 0.14915 | 4.24E-05 |
| PYGO2 | 5.10E-09 | -0.17106 | 2.72E-08 |
| RBBP5 | 2.00E-06 | -0.1108 | 6.49E-06 |
| RBBP7 | 0.001291 | 0.117276 | 0.002715 |
| RIOX2 | 1.14E-05 | 0.169288 | 3.37E-05 |
| RNF168 | 0.0004 | -0.08703 | 0.000899 |
| RNF2 | 3.36E-09 | -0.18072 | 1.90E-08 |
| RNF20 | 4.51E-05 | -0.10476 | 0.000121 |
| RNF40 | 0.007432 | -0.07071 | 0.012792 |
| RPS6KA4 | 0.008444 | 0.05617 | 0.014282 |
| RPS6KA5 | 1.80E-07 | -0.19028 | 7.09E-07 |
| RSF1 | 0.004018 | -0.11592 | 0.007443 |
| RTF1 | 0.01537 | -0.03158 | 0.024604 |
| RUVBL1 | 6.54E-12 | 0.425642 | 5.09E-11 |
| RUVBL2 | 0.003082 | 0.137109 | 0.005877 |
| SDR16C5 | 2.97E-14 | 0.777689 | 3.72E-13 |
| SETD2 | 3.86E-13 | -0.20748 | 3.58E-12 |
| SETD3 | 2.66E-18 | -0.21087 | 6.48E-17 |
| SETD5 | 0.007243 | 0.146454 | 0.012522 |
| SETD7 | 1.77E-21 | 0.429961 | 6.27E-20 |
| SETDB2 | 0.000204 | 0.14506 | 0.000481 |
| SETMAR | 4.41E-09 | -0.19761 | 2.38E-08 |
| SGF29 | 1.14E-07 | -0.24243 | 4.62E-07 |
| SIN3A | 8.73E-06 | -0.16013 | 2.59E-05 |
| SIRT1 | 2.60E-06 | -0.19482 | 8.17E-06 |
| SIRT2 | 0.009531 | 0.1058 | 0.015844 |
| SIRT6 | 6.82E-08 | -0.15137 | 2.88E-07 |
| SIRT7 | 0.000532 | -0.15132 | 0.001182 |
| SKP1 | 4.03E-14 | -0.2067 | 4.86E-13 |
| SMAD4 | 5.21E-09 | -0.18924 | 2.74E-08 |
| SMARCA5 | 0.030119 | 0.042773 | 0.046493 |
| SMARCAD1 | 0.004313 | 0.075577 | 0.007914 |
| SMYD2 | 0.000114 | 0.136731 | 0.000286 |
| SNCA | 0.000387 | 0.144905 | 0.000875 |
| SPHK2 | 2.04E-13 | -0.22256 | 2.01E-12 |
| SPI1 | 1.60E-07 | 0.221169 | 6.35E-07 |
| SUPT6H | 3.66E-05 | 0.105628 | 0.0001 |
| SUPT7L | 0.005819 | 0.091803 | 0.010431 |
| SUV39H1 | 0.000712 | 0.099683 | 0.001547 |
| SYCP3 | 2.06E-13 | -0.1932 | 2.01E-12 |
| TADA1 | 1.44E-08 | -0.20226 | 7.29E-08 |
| TADA3 | 0.015443 | -0.0706 | 0.02462 |
| TAF10 | 4.13E-05 | 0.115276 | 0.000112 |
| TAF12 | 3.27E-05 | 0.138659 | 9.03E-05 |
| TAF7 | 0.000179 | -0.09028 | 0.000425 |
| TET3 | 1.40E-14 | -0.23642 | 1.82E-13 |
| TNP1 | 0.010322 | -0.10364 | 0.017014 |
| TRIM37 | 0.002817 | 0.081952 | 0.00548 |
| TRIP12 | 1.38E-12 | 0.199905 | 1.14E-11 |
| TRRAP | 0.00293 | 0.095363 | 0.005643 |
| TWIST1 | 2.83E-18 | 0.660907 | 6.48E-17 |
| UBE2A | 0.00038 | -0.16287 | 0.000863 |
| UBE2B | 0.003065 | -0.08044 | 0.005873 |
| UBE2E1 | 2.47E-07 | -0.16452 | 9.52E-07 |
| UBR2 | 1.28E-06 | 0.118817 | 4.33E-06 |
| UBR5 | 1.68E-05 | 0.148816 | 4.81E-05 |
| VEGFA | 0.000167 | 0.256485 | 0.000409 |
| VPS72 | 0.007243 | -0.07924 | 0.012522 |
| VRK1 | 1.23E-07 | 0.375834 | 4.93E-07 |
| WBP2 | 0.000309 | -0.1279 | 0.000725 |
| WDR5B | 5.83E-06 | 0.141626 | 1.76E-05 |
| WDR70 | 0.008444 | -0.0688 | 0.014282 |
| YEATS2 | 1.36E-13 | 0.374393 | 1.40E-12 |
| ZMPSTE24 | 0.030382 | 0.077339 | 0.046713 |
| ZNF274 | 2.07E-06 | -0.10756 | 6.66E-06 |
| ZNHIT1 | 8.10E-08 | -0.11258 | 3.39E-07 |
| ATAD2B | 2.92E-05 | -0.20699 | 8.18E-05 |
| BRD2 | 7.37E-07 | 0.140758 | 2.63E-06 |
| BRD3 | 0.006263 | -0.08049 | 0.011073 |
| BRDT | 0.003561 | -0.08162 | 0.00666 |
| CBX4 | 8.17E-19 | 0.276889 | 2.12E-17 |
| CDYL | 0.003295 | -0.07394 | 0.006223 |
| CDYL2 | 0.000134 | 0.115132 | 0.00033 |
| CHD8 | 0.001572 | 0.131214 | 0.003185 |
| DPF2 | 0.021773 | 0.078573 | 0.03429 |
| GLYR1 | 2.45E-11 | -0.38121 | 1.70E-10 |
| ING1 | 0.000698 | -0.06454 | 0.001526 |
| ING2 | 0.010954 | -0.17222 | 0.017979 |
| L3MBTL1 | 0.000127 | -0.09011 | 0.000318 |
| L3MBTL2 | 0.001315 | 0.176127 | 0.00275 |
| LRWD1 | 3.69E-09 | 0.242379 | 2.05E-08 |
| MBTD1 | 4.45E-11 | -0.27752 | 2.98E-10 |
| MLLT3 | 6.56E-10 | -0.45852 | 4.05E-09 |
| MSH6 | 4.67E-07 | 0.27764 | 1.72E-06 |
| NCAPD3 | 7.64E-07 | 0.247136 | 2.68E-06 |
| NCAPG2 | 2.53E-13 | 0.260923 | 2.40E-12 |
| NTMT1 | 4.16E-07 | 0.157191 | 1.54E-06 |
| PHF13 | 0.003503 | 0.165114 | 0.006582 |
| PRDM13 | 0.007904 | -0.06578 | 0.013544 |
| RRP8 | 3.53E-07 | -0.15416 | 1.33E-06 |
| SMYD3 | 0.009155 | 0.051906 | 0.015351 |
| SPIN1 | 9.32E-21 | -0.34149 | 2.79E-19 |
| SPIN3 | 0.005178 | -0.09445 | 0.009369 |
| TAF1 | 0.001421 | -0.11012 | 0.002941 |
| TDRD3 | 3.44E-15 | -0.3614 | 4.62E-14 |
| TRIM24 | 5.43E-09 | -0.27081 | 2.81E-08 |
| TTLL12 | 0.026521 | -0.1194 | 0.041432 |
| ZMYND11 | 2.09E-07 | -0.12218 | 8.14E-07 |

^1^ Adjusted *P* values less than 0.05 was established as the significance criteria for DEGs.

##
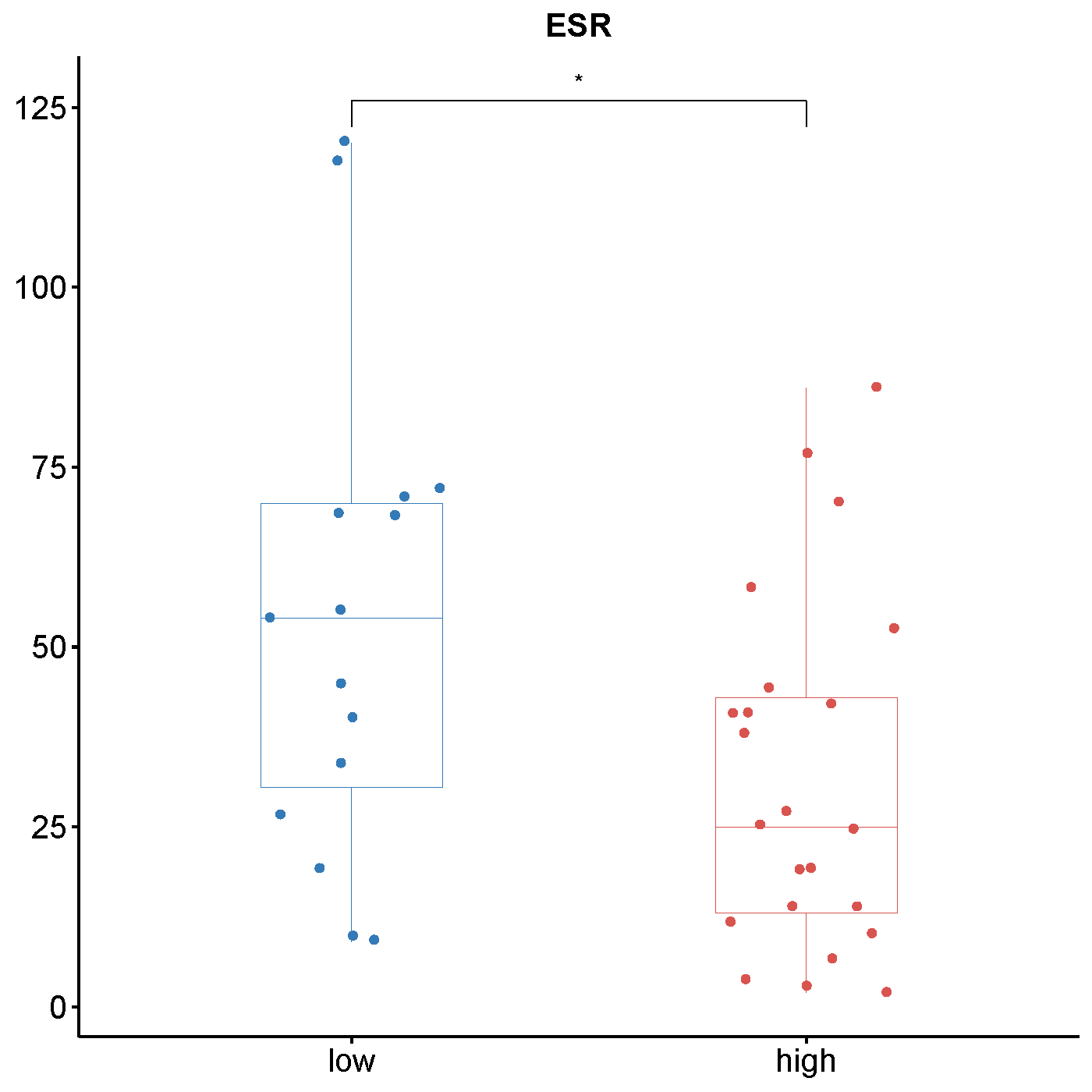


**Supplementary Figure S1.** Correlation between ESR and CAMK2D protein expression.


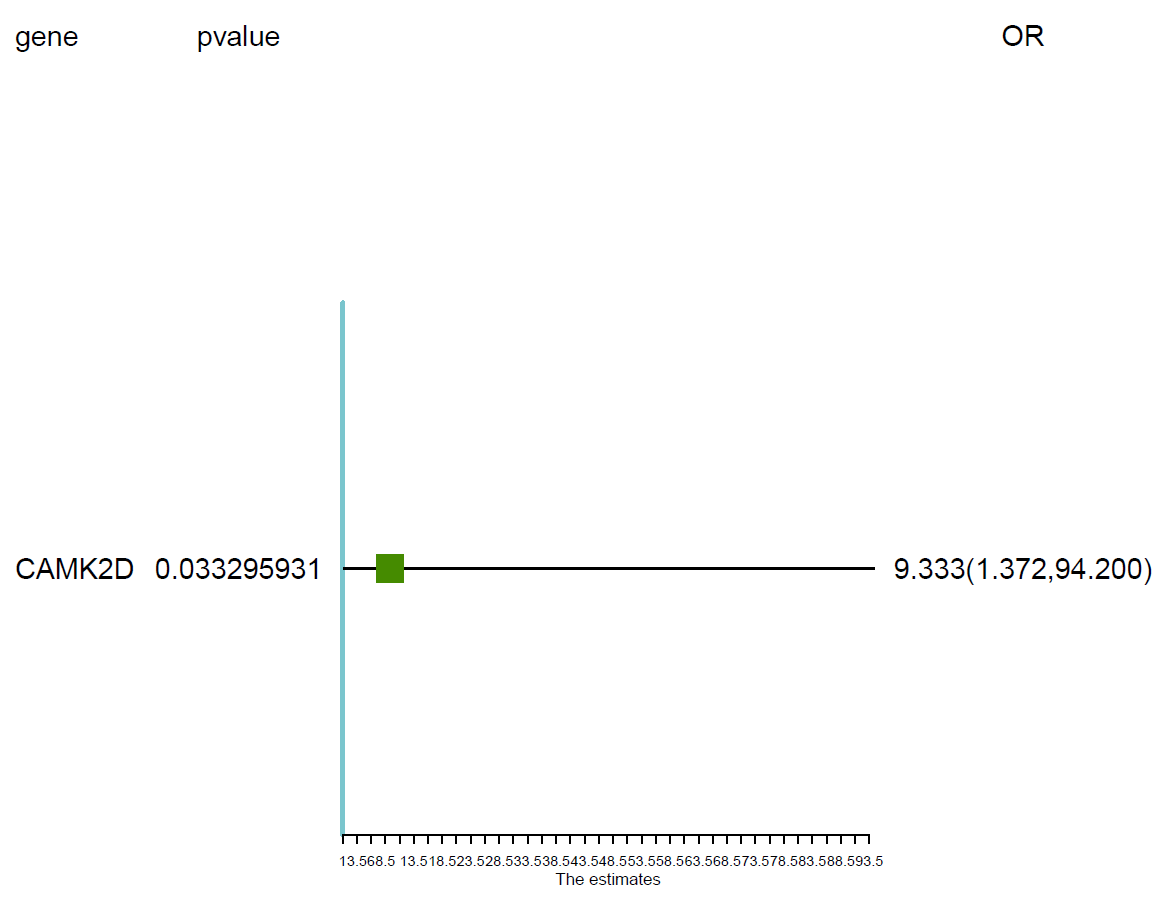


**Supplementary Figure S2.** Forest plot lines of CAMK2D protein expression level and infliximab response of UC patients in the Third Xiangya hospital by using univariate logistic regression.


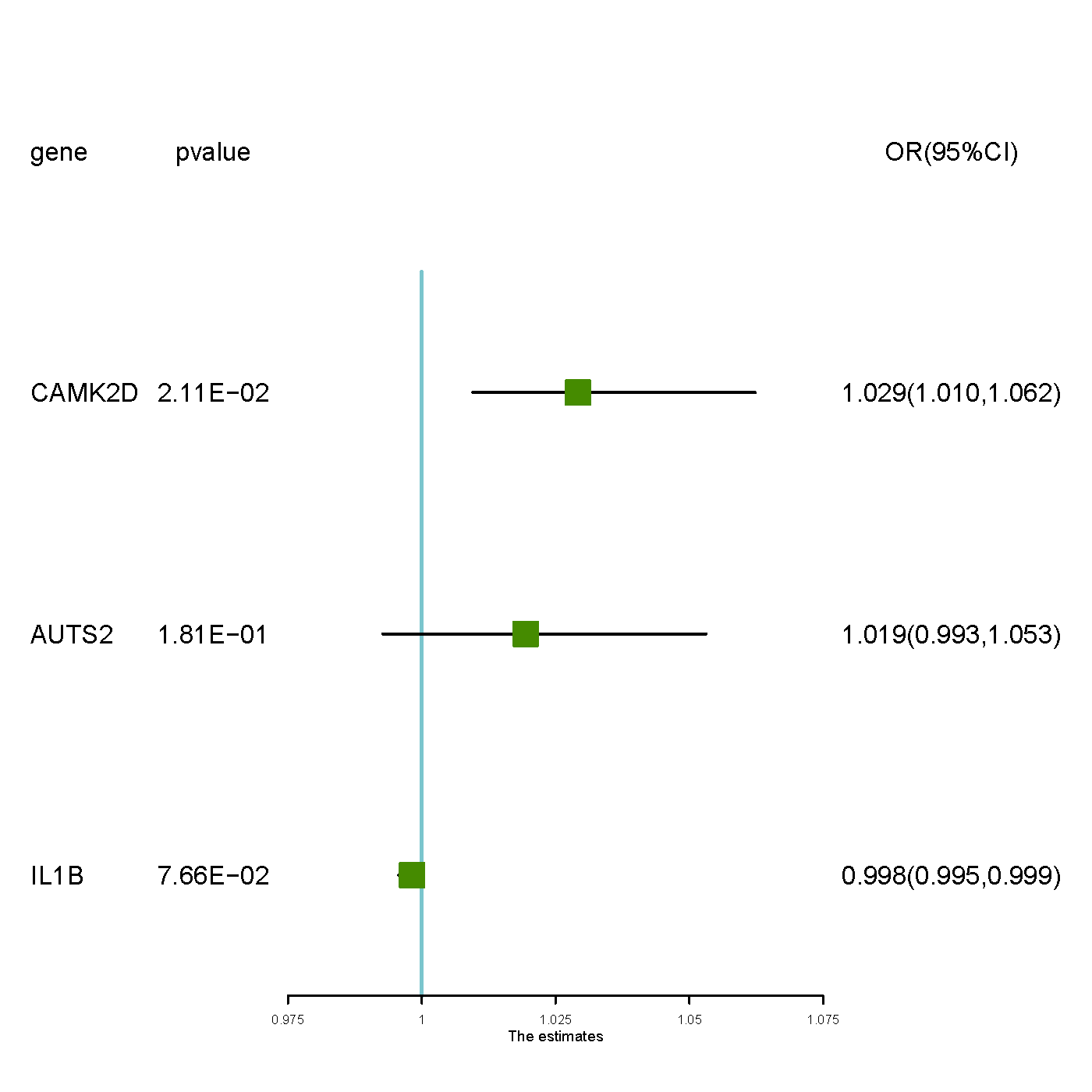


**Supplementary Figure S3.** Forest plot lines of CAMK2D protein expression level and infliximab response by using univariate logistic regression in GSE14580 dataset.
